# The Rayleigh–Lorentz invariant for superconducting resonators and optimal adiabatic qubit-information detection

**DOI:** 10.1038/s41598-021-92555-8

**Published:** 2021-07-02

**Authors:** Jeong Ryeol Choi

**Affiliations:** Department of Nanoengineering, College of Convergence and Integrated Science, Kyonggi University, Yeongtong-gu, Suwon, Gyeonggi-do 16227 Republic of Korea

**Keywords:** Mathematics and computing, Optics and photonics, Physics

## Abstract

Dynamical properties of a resonator can be analyzed using the Rayleigh–Lorentz invariant which is not an exact constant but varies more or less over time depending on variations of parameters. We investigate the time behavior of this invariant for a superconducting nano-resonator in order for better understanding of qubit-information detection with the resonator. Superconducting resonators which uses parametric resonance in a Josephson junction circuit can be utilized in implementing diverse next generation nano-optic and nano-electronic devices such as quantum computing systems. Through the analyses of the temporal evolution of the invariant, we derive a condition for optimal adiabatic qubit-information detection with the resonator. This condition is helpful for controlling the dynamics of the resonators over long periods of time. It is necessary to consider it when designing a nano-resonator used for quantum nondemolition readouts of qubit states, crucial in quantum computation.

## Introduction

Superconducting resonators^1–3^ can potentially be implemented to diverse amplification schemes for measuring weak information signals in quantum systems such as quantum computers and quantum sensors. Adiabatic readouts of qubit states in quantum computation using controllable resonators are required in optimized computing models^4–6^, while adiabatic quantum computation with a robust quantum algorithm can be achieved on the basis of the adiabatic theorem^7,8^. Due to this, adiabatic evolution of a resonator^5,9^ incorporated with nondemolition qubit information detection has attracted considerable interest in quantum-information science. It is possible to investigate dynamical properties of a superconducting resonator from the analysis of the variation of the associated Rayleigh–Lorentz adiabatic invariant^10^.

It is well known in quantum mechanics that, if the adiabatic hypothesis related to the Rayleigh–Lorentz adiabatic invariants holds, the initial eigenstate in the discrete spectrum of the Hamiltonian remains the same over time. In order to process quantum information using computing algorithms and to read out qubit-state signals, a suitable resonator is indispensable. As an implementation of quantum-information devices, superconducting qubits, such as charge qubits^11,12^, flux qubits^13,14^, and phase qubits^5,15^, are artificial two-level systems that are basic units that store quantum information. Typically, such qubits are fabricated by superconducting circuits using nanotechnology facilities. The entanglement between a qubit and a SQUID (superconducting quantum interference device) is usually used as a protocol for measuring the quantum states of the qubit^13,16^.

Adiabatic invariants that are nearly conserved quantities when the system parameters change slowly have been one of the core research subjects concerning time-varying mechanical systems. After Burgers’ pioneering work^17^ in the adiabatic hypothesis and its applications, adiabatic invariants for both nonconservative and nonlinear systems have been extensively investigated^18–20^. The reason why adiabatic invariants have become a topic of interest is that we can deduce various dynamical properties of a system from such conserved quantities, leading to deepening the understanding of the system. Indeed, adiabatic invariants are useful for characterizing quantal and photonic properties of adiabatically evolving nanosystems^21–25^.

Such Rayleigh–Lorentz invariants are not exact constants, but approximate constants under the assumption that the variations of parameters are sufficiently slow. Namely, the Rayleigh–Lorentz invariants somewhat vary with time. The study of such variation for specific systems may allow us to gain insight in understanding the underlying mechanism associated with the invariants^19^. The mechanics of such adiabatic invariance can be applied to analyzing dynamical properties of superconducting qubits in adiabatic quantum computation^8,26^.

In this work, we investigate the characteristics of the Rayleigh–Lorentz invariant of the nano-resonator system and find requirements for optimal qubit signal detection by utilizing such characteristics of the invariant. To attain fault-tolerant quantum computation, computational states should not be disturbed when we detect qubit information. Hence, wave function of a qubit state should be precluded from undergoing a decoherence-induced collapse during its measurement. In this regard, ideal qubit readout with high-fidelity is possible from a protective measurement based on the preservation of adiabatic evolution of qubit eigenstates. Protective measurement minimizes disturbance to the system and can possibly be used in situ as a standard quantum measurement with reliable precision. Based on our consequence for the condition for optimal adiabatic qubit-information detection, we will address quantum nondemolition measurement which is critical in order to extract scalable qubit information in quantum computation.

## Results

### Description of the superconducting resonator

While the picture of parametric resonance in a cavity is rich and rather complicated, the designing of superconducting resonators is flexible thanks to diverse available methods for parametric pumping^1^. Hence, the characteristics of superconducting resonators and their mathematical representation are more or less different depending on adopted models and fabrication methodologies. Moreover, for a specific resonator, the degree of approximation for its nonlinear terms in the circuit also affects the explicit form of the equation of motion for the time behavior of a flux. Throughout this work, we consider a kind of superconducting resonator that were recently proposed and analyzed by Krantz et al. for convenience^27–29^. Krantz et al. adopted quarter wavelength superconducting resonators that include a coplanar waveguide (CPW) transmission line as a practical tool for reading out qubit states. The response of this system near the resonance frequency can be modeled in terms of a parallel RLC resonating circuit. The phase difference in the SQUID is represented as $\varphi = 2\pi \phi /\phi _0$ where $\phi$ is the magnetic flux in the superconducting loop while $\phi _0$ is the magnetic flux quantum which is given by $\phi _0 = \pi \hbar /e$. Because the flux is quantized, the allowed quantities of $\varphi$ are discrete. When we describe complicated electronic circuits including Josephson junctions, we can choose either charge *q* or flux $\phi$ (or $\varphi$) as coordinate. If we choose *q* as coordinate, $\phi$ can be managed as the conjugate momentum, while *q* is regarded as momentum in the case where $\phi$ has been chosen to be coordinate. Because the flux in Josephson junctions of the SQUID exhibits nonlinear characteristics, it is favorable in this case to choose $\phi$ as coordinate^30^. The resonator acts as a parametric oscillator that can be tunable by adjusting the overall inductance (or capacitance). For some technical reasons, the modulation of frequency by a nonlinear flux-tunable inductance is preferable to tuning the capacitance^27^. The resonator can be operated by a radially oscillating small ac-flux added to a static dc-flux. In many cases, the operated angular frequency (pumping frequency) of the ac-flux is nearly twice the resonant angular frequency, $\omega _p \approx 2 \omega _r$.

**Figure 1:**
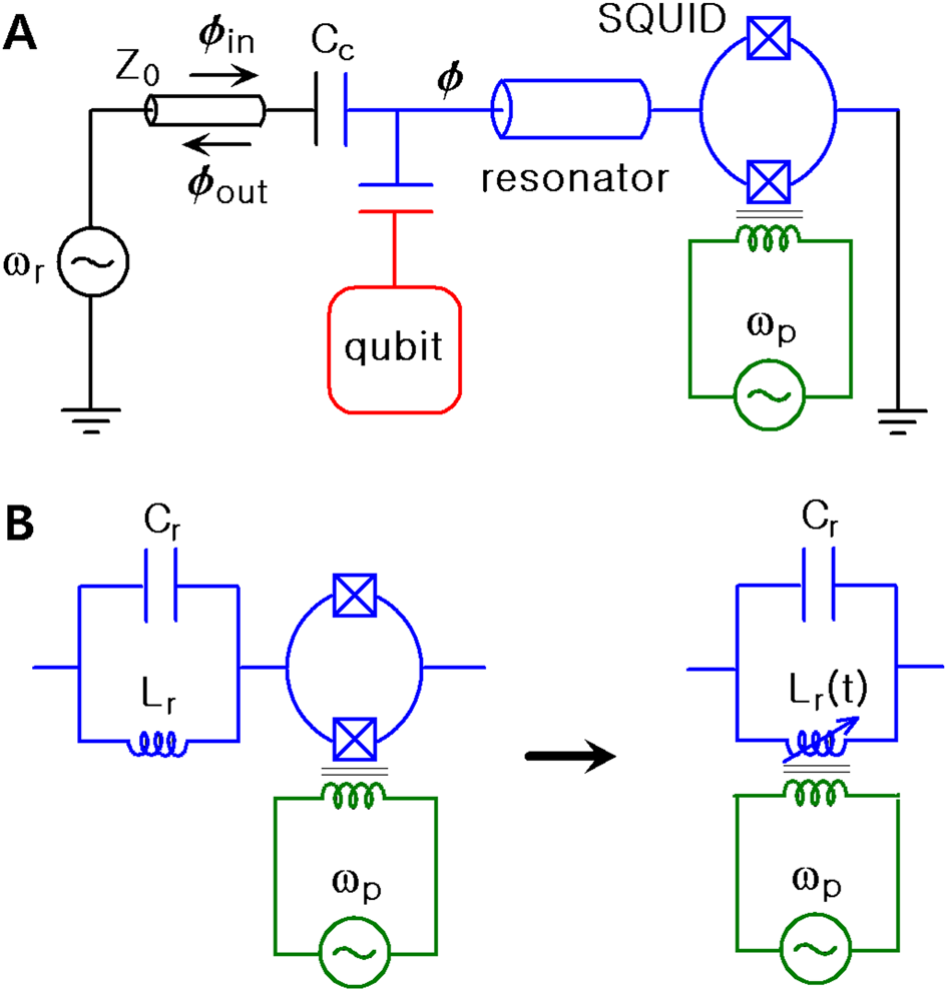
(**A**) is the schematic diagram of the CPW quarter-wavelength resonator (blue part) coupled to a qubit (red part); green part is microwave flux-pumping inductively coupled to the SQUID. (**B**) Represents that the resonator can be modeled by LC oscillator near the resonance, while SQUID gives nonlinear time-dependence of the resonator inductance $L_r$; right part of (**B**) is the equivalent diagram for this.

A schematic diagram for a tunable CPW quarter wavelength resonator considered both nonlinearity and damping is given in Fig. 1. In this case, the system is described by an extended Duffing equation which is of the form^1,29,31^

1 $$\begin{aligned} \frac{d^2 \phi }{d t^2}+(\omega _r/Q) \frac{d \phi }{d t} + \omega ^2(t)\phi -\alpha \Lambda (t)\phi ^3= \xi (t) . \end{aligned}$$

Here, *Q* is the quality factor, $\alpha$ is the Duffing nonlinear term, $\xi (t)$ is a noise or external signal, $\omega (t)$ is a time-dependent angular frequency, and $\Lambda (t)$ is a time function that is represented as

2 $$\begin{aligned} \Lambda (t) = 1- 3\lambda Q \epsilon \cos (\omega _p t)/(2\omega _r \omega _p), \end{aligned}$$

where $\lambda$ is a correction to the Duffing nonlinearity caused by a modulation of $\alpha$ through the pump, and $\epsilon$ is the strength of pumping. In Eq. (1), higher order terms have been taken into account in order to meet experimental results that exhibit pump-induced frequency shift. Nonlinearity in the system has been induced by connecting the SQUID to the cavity, whereas the damping takes place by attaching the cavity to a transmission line.

In the representation of the flux equation, Eq. (1), we have followed the convention of notations as that in Ref. 31, which are expressed in terms of $\phi$, but if we rescale the Duffing nonlinear term as $\alpha \rightarrow (2\pi /\phi _0)^2\alpha$, Eq. (1) reduces to that in Refs. 1,29. The time behavior of the Duffing coefficient $\alpha \Lambda (t)$ characterized by Eq. (2) is identical to that given in Ref. 1 under an appropriate assumption, which is $|f(t)| \ll 1$ where *f*(*t*) is a controlling field defined in that reference. The most up-to-date technique for reading qubit data with high fidelity is using the nonlinear properties of the nano-resonator coupled to the qubit system^27^.

The resonator system described by Eq. (1) can be applied to various next-generation nanotechnologies for quantum information processing. For convenience, we consider a particular case that $\omega (t)$ is given in the form^29^

3 $$\begin{aligned} \omega (t) = \left[ \omega _r^2+\epsilon \cos (\omega _p t)- \frac{\beta \epsilon ^2Q}{2\omega _r \omega _p}(1-\cos (2\omega _p t))\right] ^{1/2}, \end{aligned}$$

where $\beta$ is a dimensionless parameter. Equation (3) indicates that the oscillating frequency of the Duffing resonator is modulated in time^32^. If $\beta \rightarrow 0$, the frequency given in Eq. (3) reduces to that of Eq. (1) in Ref. 33 and/or Eq. (1) in Ref. 34, leading the system being similar to those treated in the same references. On the other hand, the last term in the bracket of Eq. (3) is the one that appeared in Eq. (1) of Ref. 35. To know how to determine various parameters in the system, refer to Ref. 27. For other models where the equation for the flux is different from Eq. (1), refer to Refs. 36,37.

Now we consider the case of a weak Duffing nonlinear term, that can be established by putting $\alpha \simeq 0$, as a solvable case. Upon this situation, the Hamiltonian describing Eq. (1) is a quadratic form and the corresponding energy can be written as (see “Methods” section which is the last section)

4 $$\begin{aligned} E(t)= \exp ({-\omega _r t/Q})\frac{\omega (t)}{\omega (0)}\left( E(0)+\frac{C\xi ^2(0)}{2\omega ^2(0)}\right) - \frac{C\xi ^2(t)}{2\omega ^2(t)}, \end{aligned}$$

where *C* is the capacitance of the resonator.

### Rayleigh–Lorentz invariant

Rayleigh^10^ discovered, for a specific time-varying system, that the quantity *I*(*t*), which is defined as

5 $$\begin{aligned} I(t) = {E(t)}/{\omega (t)}, \end{aligned}$$

almost does not vary over time, provided that the variations of system parameters are sufficiently slow. Subsequently, Lorentz rediscovered this consequence in the semiclassical regime and pronounced his discovery at the famous first Solvay Conference (for detailed reviews of this, see Refs. 19,20).

**Figure 2:**
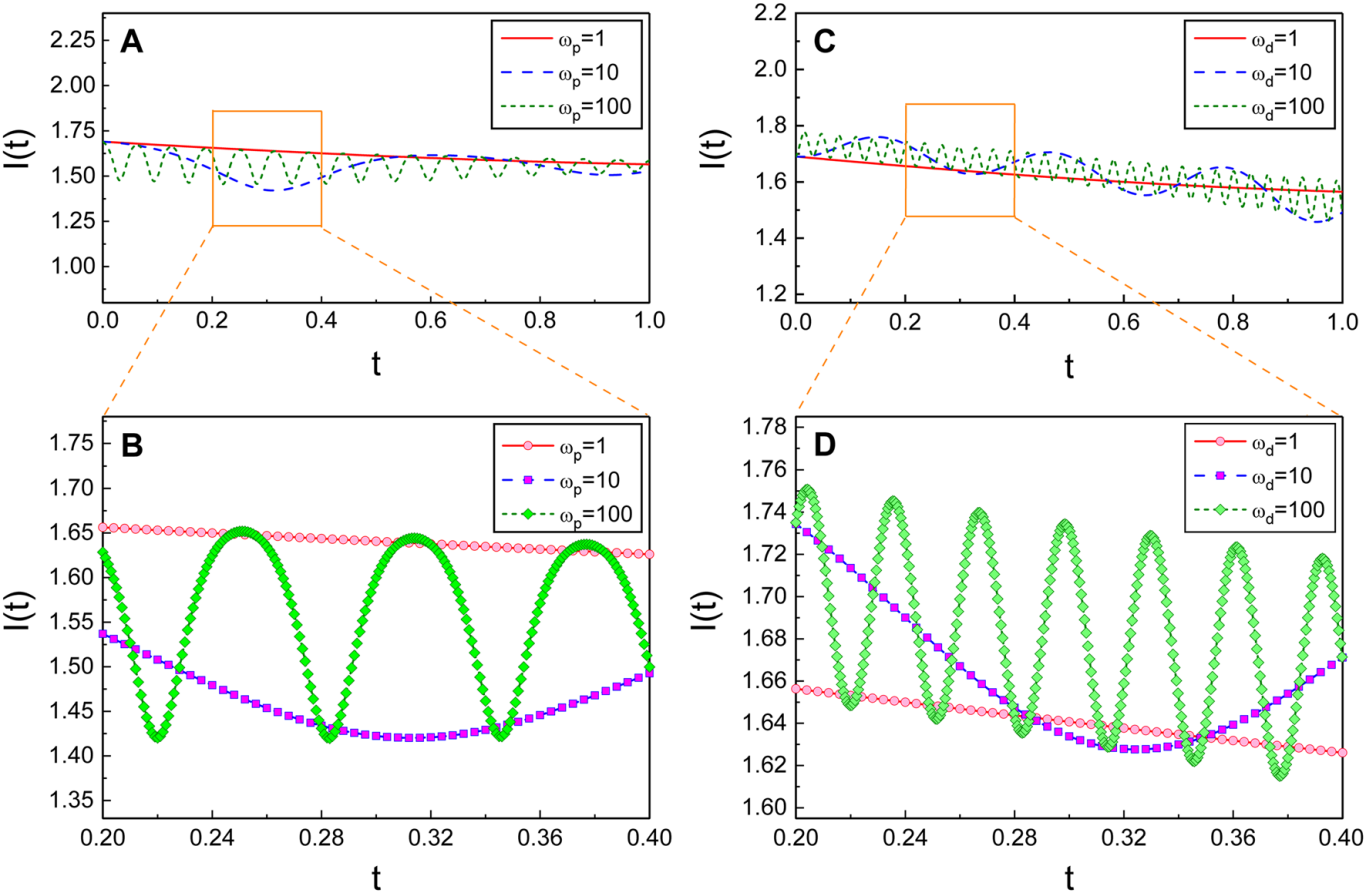
The effects of the increase of the frequencies $\omega _p$ and $\omega _d$ on temporal evolution of the Rayleigh-Lorentz invariant, where a sinusoidal noise $\xi (t) = \xi _0\cos (\omega _d t+\theta )$ has been taken. Here, $\xi _0$ is the amplitude of the external force and $\omega _d$ is the driving frequency. (**A**) is for several different values of $\omega _p$, whereas (**C**) for $\omega _d$. We have used $\omega _d=1$ for (**A**) and $\omega _p=1$ for (**C**). Other quantities that we have used are $\omega _r=0.5$, $\epsilon =0.1$, $\beta =1$, $Q=5$, $\xi _0=0.2$, $E(0)=1$, $C=1$, and $\theta =0$. (**B**) and (**D**) are enlarged plots between $t=0.2$ and $t=0.4$ for (**A**) and (**C**), respectively.

If we insert Eq. (4) in Eq. (5), we obtain the Rayleigh–Lorentz invariant of the system such that

6 $$\begin{aligned} I(t) = \frac{\exp ({-\omega _r t/Q})}{\omega (0)}\left( E(0)+\frac{C\xi ^2(0)}{2\omega ^2(0)}\right) - \frac{C\xi ^2(t)}{2\omega ^3(t)}, \end{aligned}$$

where $\omega (t)$ is given by Eq. (3). Apparently, the ratio of energy to the angular frequency is an adiabatic invariant that is useful for studying dynamical properties of the system^9^. The adiabatic invariant given in Eq. (6) is an approximate constant under the condition that the variations of parameters of the dynamical system are sufficiently slow.

**Figure 3:**
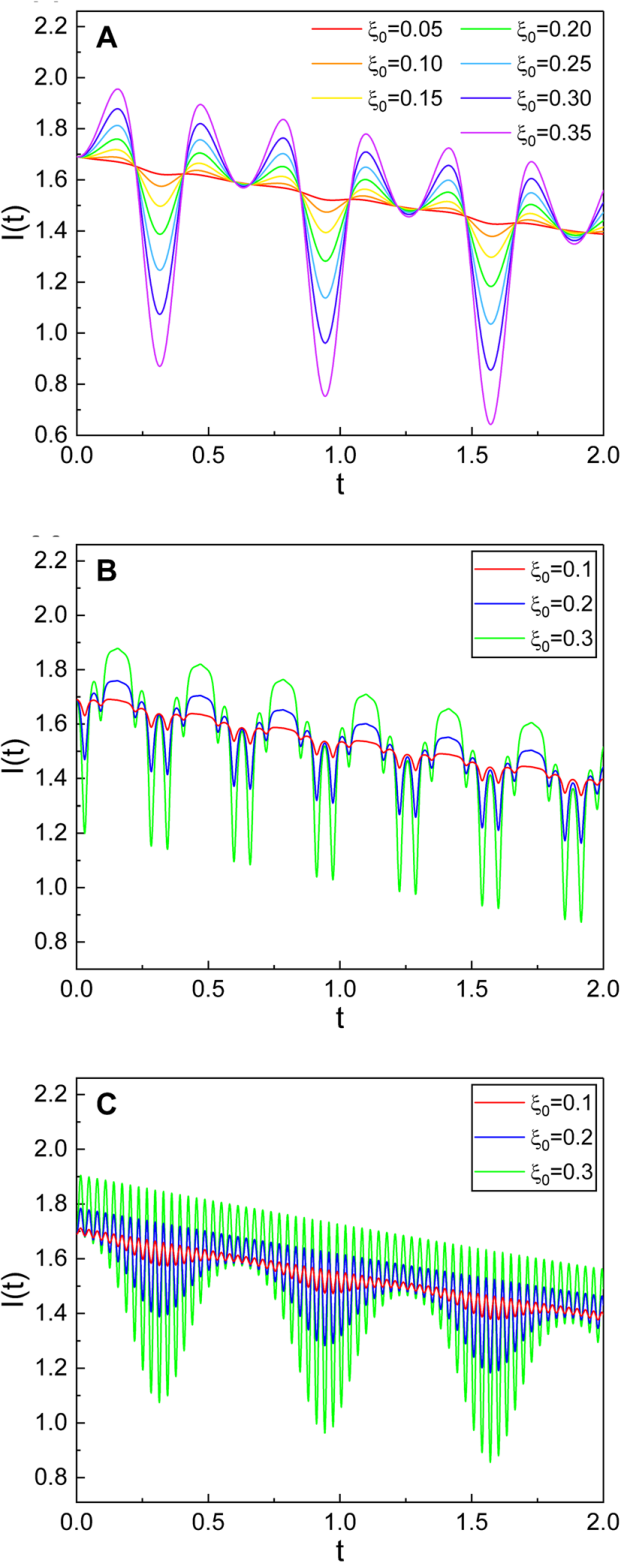
Emergence of large-scale oscillations of the Rayleigh–Lorentz invariant via the joint effects of $\omega _p$ and $\omega _d$, which is shown for several different values of $\xi _0$. The same formula of $\xi (t)$ as that in Fig. 2 is taken. The values of ($\omega _p$, $\omega _d$) that we have used are (10, 10) for (**A**), (100, 10) for (**B**), and (10, 100) for (**C**). Other values are common and given by $\omega _r=0.5$, $\epsilon =0.1$, $\beta =1$, $Q=5$, $E(0)=1$, $C=1$, and $\theta =0$.

Let us analyze the time behavior of *I*(*t*) for several particular cases. We have plotted its temporal evolution using Eq. (6) in Fig. 2 for several different choices of the value of parameters. We have chosen $\xi (t)$ as a sinusoidal form in these analyses for the purposes of simplicity. While *I*(*t*) almost does not vary for the case of small values of $\omega _p$ or $\omega _d$, it oscillates as $\omega _p$ and/or $\omega _d$ become large. S$\acute{\mathrm{a}}$nchez–Soto and Zoido discovered similar oscillations of *I*(*t*) for the systems of linear or exponentially lengthening pendulums^19^. Figure 3 shows large-scale oscillation of *I*(*t*) through the joint effects of $\omega _p$ and $\omega _d$. This figure also exhibits the fact that the amplitudes of such oscillations become high as $\xi _0$ increases. We can confirm from these analyses that, if the process of the change for the parameters of the system is too fast, *I*(*t*) would not remain constant.

### Optimal adiabatic condition

Let us deduce a useful adiabatic condition between system parameters from the formula of Eq. (6). The first term in Eq. (6) exponentially decays out as time goes by. Hence, it vanishes for a sufficient large *t* and, as a consequence, we obtain a useful parametric behavior from the remaining term, which yields at later time, as

7 $$\begin{aligned} \xi (t) \propto \omega ^{3/2}(t). \end{aligned}$$

**Figure 4:**
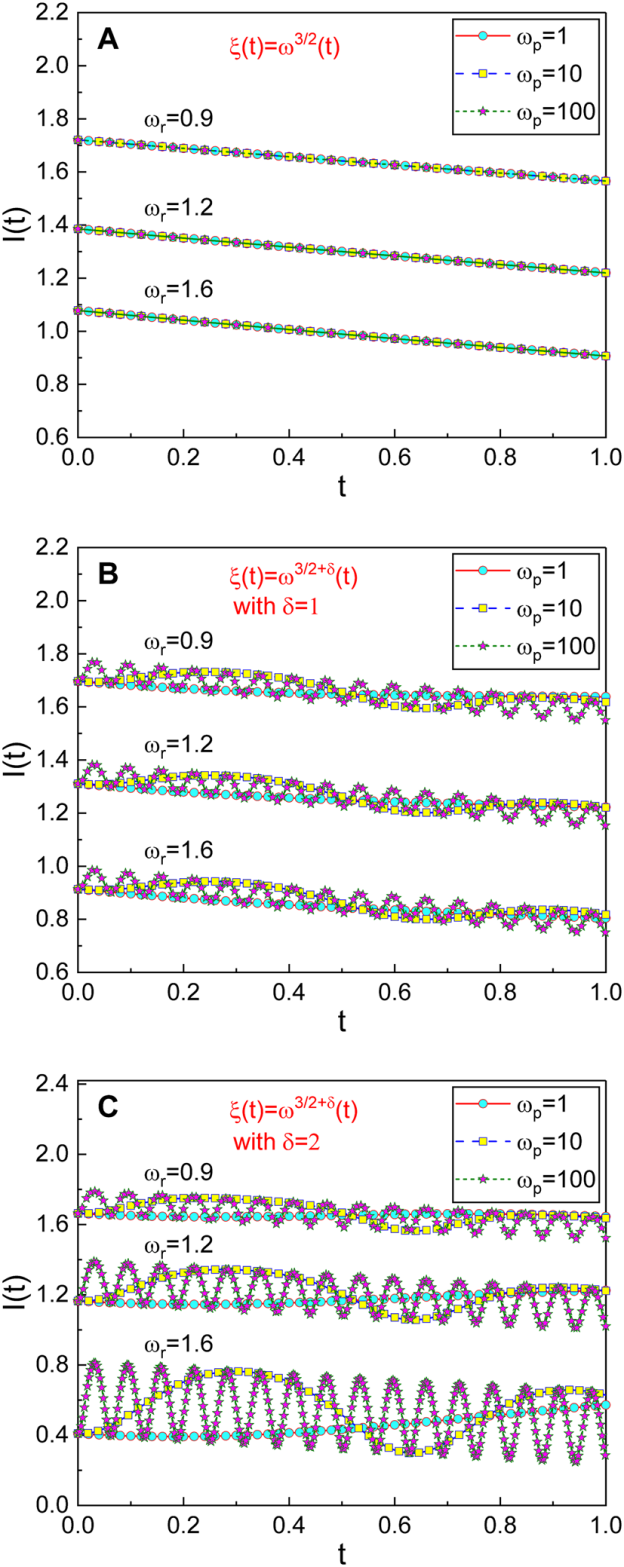
Graphical demonstration for optimal adiabatic condition from temporal evolution of *I*(*t*). Each panel is drawn for several different values of $\omega _p$ and $\omega _r$. $\xi (t) = \xi _0\omega ^{3/2+\delta }(t)$ has been taken regarding Eq. (7), where $\delta$ is a deviation from the optimal condition. Panel (**A**) is for $\delta = 0$, (**B**) for $\delta = 1$, and (**C**) for $\delta = 2$. The values of parameters that we have taken are $\epsilon =0.5$, $\beta =0.5$, $Q=10$, $\xi _0=0.4$, $E(0)=2$, and $C=1$. (**A**) shows that *I*(*t*) does not vary locally when $\xi (t)$ follows the condition in Eq. (7). If $\xi (t)$ deviates from that condition, the fluctuation of *I*(*t*) emerges (**B**,**C**).

This is the main consequence of the present work. When controlling dynamics of the resonator adiabatically^9,38^ over a long time, one should consider this relation. By comparing Fig. 4A with Fig. 4B,C (or with other previous figures), we see that the variation of the invariant is negligible in the case where this condition has been met. If we regard that one of the main problems for quantum computing is achieving high-fidelity quantum nondemolition readouts of the qubit states without measurement-induced decoherence, protective or nondemolition measurement of qubit states upon adiabatic approximation is important^39,40^. In this regard, the adiabatic condition given in Eq. (7) is useful in realizing reliable state detection with high efficiency, because it is possible to reduce the disturbance of the state substantially if such a condition holds.

Practical implementation of protective quantum measurements is based on the fact that, while we can completely describe a system using the Schrödinger wave, the quantum state in adiabatic measurement does not change throughout the experiment. Adiabatic measurement^5,6,41,42^ in addition to other adiabatic processes, such as adiabatic preparation of a state^43^ and its transfer^44,45^, enables high-fidelity operations in quantum information processing. The adiabatic condition, Eq. (7), may provide a concrete qubit-state-detection protocol which is robust not only to noise, but pulse errors as well^46^. This may open a route for a single-shot non-demolition measurement of a superconducting qubit on the basis of the adiabatic process^5^.

## Discussion

In light of the present research, the invariant plays a crucial role in investigating the dynamics of information detection of a qubit with a superconduction resonator. The invariant is approximately constant for small values of the pumping and driving frequencies. We see that the first time-dependent term of the invariant given in Eq. (6) decays exponentially. Hence, the last term in the same equation should become approximately constant over sufficiently long periods. In this way, a useful condition for adiabaticity has been found under which the invariant is approximately constant over estimated long-time scales, as shown in Eq. (7). This condition could help to control the dynamics of the superconducting resonator over long periods of time. Rigorous conservation of adiabatic invariants is requisite in order to keep the system being adiabatic during the operation of the nano-resonator^47,48^. Hence, Eq. (7) is important as a requirement for optimal qubit-information detection in protective/nondemolition adiabatic measurements^4–6,9,38–40^.

In order to prevent liable transfer of the eigenstate of the system Hamiltonian to other ones, it is necessary to preserve adiabaticity of the eigenstate. Then the collapse or entanglement of the system would not appear and, consequently, the eigenstate can be measured with a high precision. Protective measurement is possible in such a way, which helps in detecting the actual physical state characterized by the wave function for a quantum system. The temporal response of qubit readout is well-characterized and qualitatively understood from adiabatic measurement, while high fidelity readouts of a qubit state are of central importance for achieving a successful realization of quantum computers.

## Methods

The method for deriving energy expression given in Eq. (4) appears in Ref. 31. We briefly review it starting from the general representation of the energy of the system:

8 $$\begin{aligned} E(t) = e^{-2\omega _r t/Q} \frac{q^2}{2C} + \frac{1}{2}C\left[ \omega ^2(t) \phi ^2 - 2\xi (t) \phi \right] , \end{aligned}$$

where *q* is the canonical charge stored in *C*. Let us consider the classical action of the form, $J = \oint q d \phi$. Then the integration using Eq. (8) gives

9 $$\begin{aligned} J(t) = \frac{2\pi e^{\omega _r t/Q}}{\omega (t)}\bigg ( E(t)+\frac{C\xi ^2(t)}{2\omega ^2(t)} \bigg ). \end{aligned}$$

Hence, *J* has been represented in terms of *E*(*t*). Now from the relation $J(t) = J(0)$, we easily have the formula of the energy given in Eq. (4).
